# Public health policies for children and youth with special health care needs in OECD member countries and Brazil: A scoping review

**DOI:** 10.1371/journal.pone.0326645

**Published:** 2026-09-18

**Authors:** Ângela Cristina Rocha Gimenes, Beatriz Sordi Magalhães, Elenir Rose Jardim Cury

**Affiliations:** 1 Graduate Program in Health and Development in Brazil’s Center-West Region, Universidade Federal de Mato Grosso do Sul, Campo Grande, Brazil; 2 Instituto Integrado de Saúde, Universidade Federal de Mato Grosso do Sul, Campo Grande, Brazil; University of Porto Faculty of Medicine: Universidade do Porto Faculdade de Medicina, PORTUGAL

## Abstract

**Background:**

Health problems experienced by children and youth with special health care needs (CYSHCN) impose an increasingly heavy cost burden on families and countries. The vulnerability of this population group should be recognized and addressed by governments in the form of public policies.

**Objectives:**

To map the evidence on public health policies for CYSHCN in countries of the Organization for Economic Cooperation and Development (OECD) and Brazil.

**Method:**

A scoping review drawing on the PCC mnemonic strategy (Population: CYSHCN; Concept: health policies; Context: global) in accordance with the Joanna Briggs Institute (JBI) and PRISMA-ScR checklist recommendations was performed. The search expression was applied to four databases and one bibliographic index, and supplemented by a manual search. Articles and other types of literature on health treatment and recovery addressing public health policies for CYSHCN aged ≤19 years were included, irrespective of time frame or language.

**Results:**

Eleven records were included in this review. No records involving public policies were identified for most of the countries surveyed. The records selected differed in approaches and methods, irrespective of delivery level (primary, secondary, tertiary).

**Conclusion:**

Records involving national public health policies proved scarce in the countries surveyed, despite the possible existence of federal initiatives to address the health needs of CYSHCN, suggesting gaps in the provision of policies and a compounding of the vulnerability and lack of visibility of CYSHCN and their families.

## Introduction

The concept of children and youth with special health care needs (CYSHCN) was first defined in the international literature by the Maternal and Child Health Bureau (MCHB, United States), as persons who have or are at greater risk of developing physical problems or chronic, behavioral, or emotional conditions and who also require health and related services of a type or amount beyond that required by children generally [1]. This definition was adopted in the present investigation.

Not every child or adolescent with a disability [2–4] can be categorized as CYSHCN, but only those who, as per the Children with Special Health Care Needs (CSHCN) screener, experience at least one of five different health consequences: (1) use or need of prescription medication; (2) above-average use or need of medical, mental health, or educational services; (3) functional limitations, compared with peers of the same age; (4) use or need of specialized therapies; (5) treatment or counseling for emotional, behavioral, or developmental problems [5].

Health problems that manifest in early infancy or childhood and affect CYSHCN tend to persist into adulthood, requiring resource-intensive services and imposing an increasingly heavy cost burden on countries—hence the importance of devising and implementing legislation to promote programs ensuring optimal service standards for treatment of illness and rehabilitation of health, in line with the United Nations Convention on the Rights of the Child (UNCRC) [6].

Epidemiological studies have shown that 14–20% of CYSHCN in high-income countries [7–9] have some type of special health care need, representing a substantial share of the child and youth population and accounting for a large proportion of demand for specialized health services [7]. The sheer magnitude of this demand exerts a major impact on families and health care systems. In this setting, public policies play a crucial role in organizing health care systems and ensuring equitable access to comprehensive care services.

Although the importance of policies aimed at CYSHCN has been recognized, the scientific literature exhibits conceptual fragmentation and limited focus on the public policies available in different countries, particularly as regards comparative analyses of health care systems. Conducting an exploratory review of public health policies that address this population can facilitate the identification of care management models and reveal gaps in existing policies, informing the development and improvement of evidence-based strategies for CYSHCN.

The objective of this scope review was to map the evidence on public health policies for CYSHCN available in member countries of the Organization for Economic Cooperation and Development (OECD) and Brazil. This entity encompasses not only advanced economies, but also emerging partners, such as Brazil, thus serving as a forum for the discussion and development of public policies based on internationally recognized best practices, toward promoting sustainable economic growth and improving the lives of citizens.

The current investigation was prompted by an interest in identifying, tracking, and understanding public health policies and disseminating a summary of their contents, thus making these available for the development of best practices in evidence-based health care [10] and evidence-based public policies, the formulation and implementation of which should be guided by data gathered in a rigorous, and systematic manner [11]. Also, the review findings can help support future research toward boosting the development and reorientation of health care systems, ultimately reducing health complications and increasing quality of life among CYSHCN and their families.

## Method

The review was conducted in accordance with the guidelines proposed by the Joanna Briggs Institute (JBI) [12,13] and recommendations contained in the updated 2021 version of Preferred Reporting Items for Systematic Reviews and Meta-Analyses Extension for Scoping Reviews (PRISMA-ScR) (S1 File) [14,15].

### Data collection and analysis

Identification of the research gap began by searching a database to ensure the research question had not been previously answered. To this end, pertinent terms and keywords were selected. The search yielded no scoping reviews or systematic reviews on public health policies for CYSHCN.

### Scoping review guidelines

Formulation of the research question was based on the Population–Concept–Context (PCC) mnemonic strategy, where CYSHCN constituted the population, public health policies the concept, while OECD member countries and Brazil provided the context. The research question was:

### What evidence of public health policies for CYSHCN is available from OECD member countries and Brazil?

**Fig 1:**
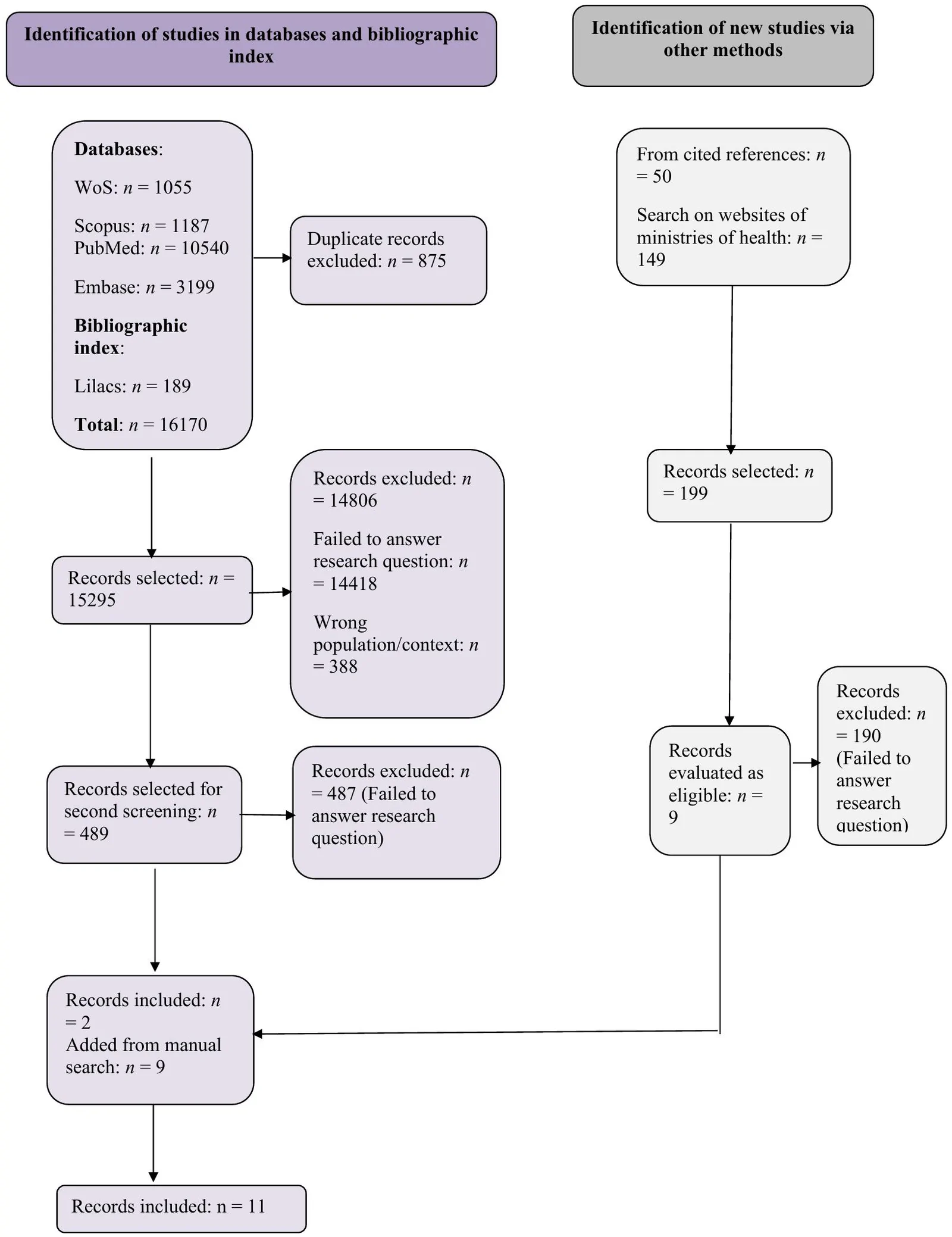
Database search, selection, and election phases of scoping review records.

### Protocol and registration

The research protocol was registered on the Open Science Framework platform (identifier 10.17605/OSF.IO/UW5BH:DOI) and published [16].

### Eligibility criteria for scoping review

Given the breadth of the research question, the scoping review corpus included not only scientific articles, but also any open-access sources addressing public health policies for CYSHCN, irrespective of language or time frame, so as to increase search sensitivity.

The following were included: documents on the programmatic provision of public health care in OECD member countries and Brazil focusing on health treatment and recovery and designed to promote comprehensive health care for CYSHCN. Authors were contacted when further information on the contents of these sources was required.

The following were excluded: scientific studies and other types of literature that failed to describe public health policies concerning aspects such as structure or levels and types of care, or that failed to provide a definition for CYSHCN. In the case of sources updating policies, plans, actions, laws, decrees, guidelines, care strategies, or lines of care, the earliest instances were excluded.

Given the cross-national nature of the investigation, the age range of 0–19 years (as per the WHO [17)] was adopted for CYSHCN, even when other ranges were included in the studies.

### Sources of information

The search strategies employed controlled vocabulary (thesaurus), combining three databases: Health Sciences Descriptors (DeCS), Medical Subject Headings (MeSH), and Embase Subject Headings (Emtree). Free terms were also used to expand the results and obtain a more sensitive, wider-reaching strategy. In addition, free-text terms were incorporated to broaden search coverage and increase sensitivity.

A health sciences librarian with expertise in database searching was consulted to design a sensitive search equation. To minimize potential search errors, the resulting strategy was independently peer-reviewed using the Peer Review of Electronic Search Strategies (PRESS) guideline checklist [18] (submitted December 12, 2022; returned December 17, 2022 [16]) (S2 File).

### Search strategies

The search strategies were first applied on December 22, 2022, and reapplied after update on October 5, 2024, in the same databases and bibliographic indexes. In the updated search, a temporal filter was used to identify studies published from 2023 onwards, so as to incorporate more recent evidence. Manual searches were also reviewed and updated in October, 2024 (S1 Checklist. Manual search of websites).

The search expressions are available from the following repositories:

- Embase: https://doi.org/10.1079/searchRxiv.2023.00314;
- PubMed: https://www.cabidigitallibrary.org/doi/10.1079/searchRxiv.2023.00307;
- Scopus: https://doi.org/10.1079/searchRxiv.2023.00315;
- Web of Science: https://doi.org/10.1079/searchRxiv.2023.00316;
- Lilacs: https://doi.org/10.1079/searchRxiv.2023.00317;
- Medical Literature Analysis and Retrieval System Online (Medline), via PubMed:https://www.cabidigitallibrary.org/doi/10.1079/searchRxiv.2023.00307.

The manual search performed on websites is available in the S1 checklist.

### Selection of sources of evidence

The retrieved records were imported into Rayyan software (Qatar Computing Research Institute) [19] and subsequently analyzed by two independent reviewers tasked with screening the search output. For the initial screening of articles, titles and abstracts were rated as ‘Yes’, ‘No’, or ‘Maybe’, without using filters. When titles and abstracts proved insufficient for reaching a decision, the articles were marked for full reading.

The second screening entailed reading the full texts. Titles, decisions (include/exclude), reasons for exclusion, and comments were compiled in an Excel spreadsheet. Disagreements were resolved through consensus. Sources deemed relevant were selected from the reference lists of articles and subsequently retrieved.

A calibrating, pilot inter-rater test was performed for 30 items of evidence using Cohen’s kappa coefficient, yielding a value of 0.7826 (*p* ≤ 0.001), indicative of excellent agreement (0.40–0.59: reasonable; 0.60–0.74: moderate; ≥ 0.75: excellent) [20].

### Data extraction

Following the selection of articles, an Excel spreadsheet, whose contents were adapted from the JBI method, was created to collate information on titles, authors, methods, health policies or legal documents cited, target populations, countries, years, objectives (S1 Table), and salient points of public health policies (S2 Table) [21,22].

A pilot test of the data extraction spreadsheet was performed by two team members, on three sources. The first part of the pilot test consisted of a meeting of roughly one hour to present the spreadsheet and expound on the purpose of each item to be extracted, as well as to clear up any queries that might arise. The second part entailed analyzing three randomly selected records and the data extracted from them. This step yielded an inter-rater agreement level of 78.8%, exceeding the expected level of 75% established when designing the research protocol. The rate obtained confirms that all data extracted were relevant.

A descriptive text was composed reporting the health policies found in the review, together with authors and other data of relevance for the research objectives and question. A summary of the main results was prepared, providing an overview of the health policies found and knowledge gaps detected.

### Summary of evidence

Results providing answers to the research question were expressed as figures, tables, and a descriptive text.

### Ethical issues

Given the study was a scoping review, the need for submission to a research ethics committee was waived. However, regarding the analysis and sharing of results, the study complied with resolution 466/2012 of the Brazilian Health Council [23].

## Results

Of the eleven records included in this review, two were retrieved through the systematic database search and nine via manual search (Fig 1). These numbers suggest not only that public health policies for CYSHCN are scarce, but also that manual searching is important for review studies on public policies, given that some laws, resolutions, and even discretionary measures by public management agents can only be found on institutional websites (S1 Table). Relevant points, years of publication, and countries of origin of these records are listed in S2 Table.

Records also differed by type and included a literature review, four technical guidelines, two websites, a guide, two policy guidelines, and a descriptive article. This heterogeneity, coupled with discrepancies observed in the records concerning public health policies for CYSHCN, hampers cross-national comparisons.

Only 11 records of public health policies for CYSHCN were found, originating from seven of the 39 countries investigated (Fig 1): United States (3 records), Chile and Germany (2 each), Poland, United Kingdom, Brazil, and Italy (1 each)—hence, record originated predominantly from the American continent.

S3 Table summarizes the core features of these policies (policy identification, organizational model, care coordination mechanisms, funding mechanisms, and degree of integration), allowing different systems to be compared in terms of similarities, specificities, and strategies for the provision of comprehensive care.

## Discussion

Countries where ministries of health do not play a leading role often have concomitant province- or state-level health care subsystems often issue differing public health recommendations, with consequent inconsistencies across social or racial groups, potentially aggravating the vulnerability of children, youth, and older adults [24]. Therefore, the present investigation focused on country-level public policies. However, this methodological choice may have led to underreporting of regional public policies of decentralized health care systems, impacting the number of records found.

No records of public health policies for CYSHCN were found for 32 (82%) of the 39 countries surveyed. A number of factors might explain this absence, including childhood and adolescence being assumed as normally healthy periods in human life, or a lack of consensus on the terminology and definition of CYSHCN, with consequent confusion with other population segments, such as children and adolescents with disabilities. In such cases, health care provision has probably been delineated through other public health policies—*e.g.*, those aimed at chronic conditions in adults [25] or providing for acute or prevalent conditions affecting children and adolescents, [26,27] with no regard to CYSHCN specificities.

Public health policies for CYSHCN should cater for the range of needs of this population, and at different levels of complexity (primary, secondary, and tertiary).[28,29] Achieving meaningful results hinges on effective organization, with primary care playing a crucial role as the coordinator of this process [30]. Unfortunately, care offered to this population has typically been limited to secondary and tertiary care services.[31,32]

This observation is corroborated by studies reporting that the social health care network for CYSHCN is typically restricted to tertiary level (referral hospitals), owing to inaccessibility of these users to primary care. These circumstances fragment healthcare and increase the risk of social vulnerability [28,33,34].

In Germany, for instance, medical-social monitoring, enshrined in law §43 Abs.2, Sozialgesetzbuch (SGB)V, is available in the transition from inpatient to outpatient care for children and adolescents with serious chronic conditions, but these services are provided only by case managers designated by health insurance providers, not in connection with primary care, possibly reflecting structural features of the country’s health care system [35,36].

Brazil has a policy that, although similar to its German counterpart, is not regulated by law as in Germany. This renders health care provision to CYSHCN in Brazil vulnerable to governmental changes and shifting economic priorities. The objective of the Brazilian policy is to promote safe, responsible hospital discharge, requiring this phase to be mediated by a multidisciplinary team (termed Long-Term Hospitalization Management – Geinpro), responsible for discharge planning, partnerships between the various different levels of care, caregiver qualification, risk assessment of the family´s area of abode, and scheduling of pre-admission home visits [37].

Provisions for the transition from in-hospital to outpatient services also entail not only coordinating services and professionals across the different levels of care (primary, secondary, tertiary), but also planning for the patient’s family, whose members have to cope with adjusting to this transition. This approach helps promote reintegration into social and family environments, while improving the quality of life of these children and adolescents, as well as that of their families.

This type of support is doubtless vital when families have to adapt to new routines. Studies have shown that, irrespective of country or culture, the primary family caregiver is typically the mother or a grandmother. While endeavoring to shape a social support network, they also reshape their daily lives, give up paid work, seek financial assistance and other resources for care provision, search for places in daycare centers and schools, and learn how to deal with medication and technologies.[38,39]

Provision of services at all levels of care, to ensure comprehensiveness in public health care for CYSHCN, was found to be available in the United States, United Kingdom, Italy, Chile, and Brazil. Chile, however, was the only country to have a comprehensive public health care model specifically designed for this population.

The Chilean comprehensive care model for CYSHCN has four components: person-centeredness, stratification by risk level, continuity of care, self-management, and shared decision-making. This requires health care teams, both at primary and secondary level, to align their actions and provide these patients and their families with coordinated care. At admission to primary care, Chilean CYSHCN undergo comprehensive health check-ups (by a physician and a nurse), nutritional evaluation (dietitian), psychosocial assessment (psychologist and social worker), and dental check-up (dentist and oral health technician). A home visit is also scheduled (nurse, dietitian, and social worker) [30,31].

In Brazil, also with a public health care system, policies aimed at children and adolescents or for chronic conditions do not provide guidance on the health management of CYSHCN by primary health care services, concerning aspects such as identification of these conditions or definition of therapeutic approaches and monitoring of care coordination, even though these policies state the need for articulation and comprehensiveness of care in conjunction with the family (through a Customized Therapeutic Project) or provide guidelines for referrals, when needed, for health surveillance in cases of chronic diseases [27,40–43].

Identification of weaknesses in the care delivered to CYSHCN in the primary setting (*e.g.*, functional barriers to access, insufficient bonding between families and primary care services, poor support, shortage of professionals and specialized services, lack of knowledge on the rights of CYSHCN, and disorganized care flow) can provide elements for reorganizing primary care services, improving effectiveness, reducing red tape, and designing appropriate guidelines for better care flow and building of lasting bonds with families.[44,45]

In the United States, concerns with the emerging, substantial population of CYSHCN have led to the creation of the Title V Maternal and Child Health Services Block Grant, a program to provide coordinated, family-centered care and promote development of community service systems by earmarking at least 30% of federal Title V funds each year, with the requirement that every US$4 of federal Title V funds be matched with at least US$3 from states.[46,47] For 2026, the federal budget was estimated at US$210,118,639, in addition to funds earmarked by states [48]. Such high investment illustrates the country’s concern with this issue, as also evidenced by the fact that the United States ranks first not only in the output of scientific publications on childhood chronic conditions, [49,50] but also in the enactment of legislation benefiting children.

Securing support for health care for CYSHCN is critical, but because decisions on eligibility under Title V are left to the discretion of each American state, different classifications and definitions of CYSHCN coexist in the country. Furthermore, depending on the funding available for programs and services, agencies are often faced with differing eligibility criteria, potentially resulting in the exclusion of some CYSHCN, as well as in discrepancies in service provision across states, [51] precluding effective interaction and integration.^52^

In the United States, the Maternal and Child Health Bureau took the initial steps toward a national research agenda on health care systems for CYSHCN [52]. A strategy called “Blueprint for Change: Guiding Principles for a System of Services that Meet the Needs of CYSHCN and Their Families” was thus developed [53].

The strategy is designed to allow CYSHCN to enjoy a full life from childhood to adulthood, with dignity, autonomy, independence, and active participation in their communities, enabled by a system which supports their social, health, and emotional needs. The four objectives of the strategy are health equity; family and child well-being and quality of life; access to services; and financing of services.[53,54].

Brazil [37] and Germany [35,36] prioritize similar policies of dehospitalization. Poland guarantees additional health services through health insurance companies [55]. The United States provides financial input, despite differences in coverage across states and health insurance companies [48]. Comprehensive and coordinated health services are outlined in policies of other countries: in the United Kingdom, continuous care is guaranteed with resources, planning, coordination, and support in the assessment of needs of CYSHCN, [56] and Italy has implemented a model integrating hospital and territory to ensure provision of universal assistance [57]. These policies, however, do not provide clear guidelines for the therapeutic progression from primary to secondary and tertiary care, thus failing to fully address the comprehensiveness and coordination of care required by this heterogeneous population.

This fragmentation of care provision compromises comprehensiveness and access, aggravating the vulnerabilities of CYSHCN. Barriers to access can also be exacerbated by unfavorable social determinants of health, including unemployment, lack of basic sanitation, violence, and environmental pollution.

Public health policies that neglect these determinants possibly draw on a biomedical model (focused on body, disabilities, physician, and medicalization) rather than a social model (focused on social and structural barriers), and care and health outcomes may vary according to the model adopted, compromising comprehensive health care when the social, psychological, and territorial factors experienced by families are disregarded.

A few pragmatic questions can serve to illustrate these inequalities: How would a CYSHCN living in a rural area with no basic sanitation gain access to dehospitalization services? How would health professionals reach CYSHCN living in a remote or violent area? How would a CYSHCN of low socioeconomic status have adequate and safe transportation to a therapy center? How should their families adapt their homes to receive equipment such as hospital beds? These and other gaps in public policies may worsen health inequalities among CYSHCN.

Only by recognizing the full range of needs, and implementing measures capable of overcoming geographical and structural barriers, can health care systems provide more equitable, continuous, and responsive care to CYSHCN, helping reduce inequalities and strengthening the comprehensiveness of care. Telemedicine constitutes one of the resources capable of mitigating difficulties in access to health services, as demonstrated during the COVID-19 pandemic, with a potential to reduce disparities among CYSHCN. This approach is particularly beneficial for those living in rural areas and individuals who, owing to medical complexities, are frailer and require special transportation [58–61].

### Limitations

Limitations of this study should be taken into account when interpreting its findings. The absence of a standardized international definition of CYSHCN and the terminological diversity observed across countries can lead to omissions and hinder consistent identification of policy reaches. Heterogeneity in the gray literature, indexing errors, or publication biases may also have contributed to relevant documents being missed, despite the breadth of the search strategy.

Searches on the websites of ministries of health often encounter structural barriers, including a lack of updated, centralized repositories, data dispersion across subportals, and a variety of formats, all of which may have limited the coverage of policies, especially when recently issued.

Lastly, by focusing on federal policies, this study may have failed to capture relevant subnational initiatives, particularly in decentralized health care systems. Future research should explore state and regional analyses to deepen the understanding of institutional arrangements and local dynamics that shape the provision of care for CYSHCN.

It is important to acknowledge that the absence of records identified for a given country does not necessarily indicate the absence of public policies. At least three distinct scenarios may underlie a “no records” result: (*i*) genuine absence of a formal, country-level policy addressing CYSHCN; (*ii*) existence of relevant policies that our search strategy failed to capture—*e.g.*, owing to indexing gaps, language barriers, or the structure of government websites; and (*iii*) policy content embedded within broader child-health, disability, or chronic-disease frameworks without an explicit CYSHCN designation, and therefore not identifiable as such under our eligibility criteria. It was not possible to fully distinguish these scenarios on a country-by-country basis, a fact that constitutes an important limitation in interpreting the finding that 32 of the 39 countries surveyed had no identified records.

## Conclusions

The findings reveal a set of critical dimensions permeating the design and implementation of public health policies aimed at CYSHCN. Particularly salient among these dimensions are the invisibility of this population in health care systems, insufficient financial support, care fragmentation, limited coordination between levels of care, health inequities, and challenges related to professional training.

The invisibility of CYSHCN is evidenced by both the scant epidemiological data and limited discussion of the issue in public and institutional forums. This invisibility contributes to a lack of priority being given to the needs of CYSHCN and their families on government agendas, aggravating inequalities in access to services and in allocation of resources. The absence of robust information systems also makes it hard to estimate the true size of this population and its specific needs.

Another relevant challenge is the fragmentation of care and the limited coordination and comprehensiveness of interventions, especially in contexts where national guidelines or well-defined institutional arrangements are lacking. The absence of integration between levels of care compromises care continuity, increasing the burden on families and aggravating social and economic vulnerability.

Health inequities associated with complex chronic conditions among CYSHCN also stem from insufficient funding and heterogeneity in the training of health professionals to deal with complex chronic conditions in childhood and adolescence. Unavailability of specific training and absence of structured care models limit the capacity of health care systems to cater equitably and effectively to the needs of this population.

Lastly, the findings highlight a lack of international consensus on the definition of CYSHCN—a cross-cutting challenge in this area. Conceptual inconsistencies across countries, regions, and health care systems hinder the comparability of studies, the development of epidemiological indicators, and the design of mutually aligned public policies. These important aspects, however, do not remove the need to reflect on whether adopting an international consensus is actually feasible or whether approaches adapted to national and cultural contexts would be more effective in guiding health policies and practices.

The materialization of specific care for CYSHCN is today a global imperative, and cross-national entities, in joint efforts with governmental and professional authorities, are faced with this emerging challenge. Achieving this goal will entail promoting and (re)organizing health care networks, ensuring sources of financing, and designing public health policies and action plans capable of guaranteeing the right to health and the full development of these children and youth under the auspices of a comprehensive, coordinated health care model.

## Supporting information

S1 Table Studies included in scoping review. (DOCX)

S2 Table Key points of public health policies. (DOCX)

S3 Table Key features of public health policies. (DOCX)

S1 File PRISMA-ScR. (DOCX)

S2 File PRESS. (DOCX)

S1 Checklist Manual search of websites. (DOCX)
