# Supplementary material for: Public health policies for children and youth with special health care needs in OECD member countries and Brazil: A scoping review
Source: PLoS One. 2026 Sep 18;21(9):e0326645. doi: 10.1371/journal.pone.0326645 (PMC13588354; doi:10.1371/journal.pone.0326645)
Supplement: Supplementary file 1 [file pone.0326645.s001.docx]

S1 Table. Studies included in scoping review.

| **Title** | **Authors** | **Method** | **Legal basis cited and year** | **Target public** | **Country and year of publication** | **Objectives** |
| --- | --- | --- | --- | --- | --- | --- |
| Sozialmedizinische Nachsorge für schwer und chronisch kranke Kinder: Eine neue Leistung der Krankenkassen | A. Podeswik; F. Porz; K. Groeger; U. Thyen | Literature review | Social-medical support.  Section 43, paragraph 2, of Book V of the Sozialgesetzbuch (German Social Code), 2008. | Children and adolescents with serious chronic diseases | Germany, 2009 | Bridging gaps in transition from inpatient to outpatient care. |
| Norma Técnica para la Supervisión de Salud Integral de Niños y Niñas de 0 a 9 Años en la Atención Primaria de Salud – Actualización 2021 (V. 3) | Ministerio de Salud | Technical guidance | National Child Health Program: Approach for children with special health care needs | Children up to age 9 with special health care needs | Chile, 2021 | Monitor growth and development of child population through systematic contact of health teams with children and their families. |
| Children and Youth with Special Health Care Needs (CYSHCN) | Health Resources & Services Administration | Website | Blueprint for Change:  Title V Maternal and Child Health Services Block Grant | CYSHCN and their families | United States, 2024 | Improve health and quality of life of CYSHCN. |
| Uprawnienia szczególne | Narodowy Fundusz Zdrowia | Website | Na podstawie ustawy z dnia 27 sierpnia 2004 r. (Law of 27 August 2004 on publicly funded health services.)  Regulation of the Ministry of Health, issued 6 November 2013, guaranteeing dental treatment services. | Children and adolescents with severe disabilities | Poland, 2004 | – |
| Orientación técnica para el manejo integral de niños, niñas y adolescentes con necesidades especiales de atención en salud (NANEAS) | Ministerio de Salud | Technical guidance | 1. Explicit Health Guarantees for CYSHCN.  2. Respiratory health programs:  2.1. Non-Invasive Ventilatory Assistance Program.  2.2. Invasive Ventilatory Assistance Program.  2.3. Home-delivered oxygen therapy program.  3. “Ricarte Soto” Act ( (Law 20 850, undated). | Health professionals and multidisciplinary teams at all levels of care who cater to CYSHCN, their families, and caregivers. | Chile, 2022 | Orient PHC teams in the provision of comprehensive care to CYSHCN and in monitoring their health, for improved quality of life of these patients and their families. |
| Title V Maternal and Child Health Services Block Grant to States Program Guidance and Forms for the Title V Application/Annual Report | Health Resources & Services Administration | Guidance for annual report | Title V Maternal and Child Health Services Block Grant | CYSHCN up to age 21 | United States, 2023 | Provide and promote coordinated family-centered and community-based care |
| Children and Young People’s Continuing Care National Framework | Department of Health and Social Care | Technical guidance | Health and Social Care Act 2012; Children and Families Act 2014 | Clinical commissioning groups (managers responsible for achieving best possible health outcomes for their local population), local authorities, health and wellbeing boards, healthcare professionals. | United Kingdom, 2016 | Provide guidance to clinical commissioning groups in assessing needs of children and youth with complex needs |
| Progress, Persistence, and Hope: Building a System of Services for CYSHCN and Their Families | Michael D. Warren, Sarah E. McLellan, Marie Y. Mann, Joan A. Scott, Treeby W. Brown. | Technical guidance | Blueprint for Change: Guiding Principles for a System of Services that Meet the Needs of CYSHCN and Their Families | CYSHCN | United States, 2022 | Showcase the historic leadership of the Maternal and Child Health Bureau in advancing a system of services for CYSHCN and introduce the Blueprint for Change. |
| Linee di indirizzo per la promozione e il miglioramento della qualità, della sicurezza e dell'appropriatezza degli interventi assistenziali in area pediatrico-adolescenziale | Ministero della Salute | National guideline, comprising 10 lines of action | Piano Nazionale Cronicità (PNC), 2016 | Children with complex chronic diseases | Italy, 2017 | Ensure multiprofessional care centered on patient and the whole family unit, to improve quality of life, promote autonomy and social inclusion, reduce the risks of complication, and ensure effective prevention, including vaccination.  Ensure a global approach to complex chronic diseases, taking into account both health and social aspects. |
| Desospitalização: reflexões para o cuidado em saúde e atuação multiprofissional | Ministério da Saúde | Guideline | Ordinance 2029, 24 August 2011.  Ordinance 1208, 18 June 2013.  Política Nacional de Humanização.  Cadernos de Atenção Domiciliar. | Children with complex chronic conditions | Brazil, 2020 | Plan for actions involving dehospitalization, responsible discharge, and transition from safe care to other health services. |
| Entlassung von Kindern mit komplexen Versorgungsbedarfen von der neonatologischen Intensivstation | Stephanie Ballmann | Descriptive article | §43 Abs.2, Sozialgesetzbuch (SGB)V | Children with complex needs | Germany, 2024 | Describe the model of aftercare provision shared between outpatient hospitalization sectors, ensuring outpatient care and early discharge. |
