# Supplementary material for: Public health policies for children and youth with special health care needs in OECD member countries and Brazil: A scoping review
Source: PLoS One. 2026 Sep 18;21(9):e0326645. doi: 10.1371/journal.pone.0326645 (PMC13588354; doi:10.1371/journal.pone.0326645)
Supplement: Supplementary file 2 [file pone.0326645.s002.docx]

S2 Table. Key points of public health policies.

| **Title, country, year** | **Key points** |
| --- | --- |
| Sozialmedizinische Nachsorge für schwer und chronisch kranke Kinder: Eine neue Leistung der Krankenkassen.  Germany, 2009. | Bridges gaps in the transition from inpatient to outpatient care, ensures communication and coordination, and relieves the burden on patients' families through external support. Begins at the end of the hospital stay, helps manage the transition home, organizes and coordinates outpatient therapies, visits, and training, as well as rehabilitation services. Aftercare team visits patient on the ward, then makes home visits and accompanies them to the treatment facilities. Follow-up care is carried out through case management action plan. |
| Norma Técnica para la Supervisión de Salud Integral de Niños y Niñas de 0 a 9 Años en la Atención Primaria de Salud – Actualización 2021 (V. 3).  Chile, 2021. | Flow of care for CYSHCN aged 0 to 9 years comprises two stages: (1) admission to primary care, with evaluation shared by a physician and nurse, and subsequent referral to nutritional admission evaluation by a dietitian, followed by evaluation shared by a psychologist and a social worker; (2) health monitoring activities, where health status is monitored and adjustments are made to health plan established at admission time. These activities include health examinations, nutritional consultations, team meetings and coordination across the three levels of care. |
| Children and Youth with Special Health Care Needs (CYSHCN)  United States, 2024. | Blueprint for Change: a national framework for a system of services for CYSHCN so they can enjoy full lives and thrive in their community from childhood through adulthood, with support for their families and their social, health, and emotional needs. The program aims to ensure dignity, autonomy, independence, and active participation in their communities, and involves six indicators of a coordinated, comprehensive family-centered system.  Title V:  Strengthens service delivery capacity of state-level public health agencies, health care providers, and organizations that serve CYSHCN. |
| Uprawnienia szczególne.  Poland, 2004. | Primary health care services (physicians, nurses, midwives), hospital services, specialized outpatient services (outpatient specialists), pharmaceutical services provided in pharmacies, medical rehabilitation services (according to medical indications, without limit), and general anesthesia if required for dental treatments. |
| Orientación técnica para el manejo integral de niños, niñas y adolescentes con necesidades especiales de atención en salud (NANEAS).  Chile, 2022. | 1. Comprehensive care model for CYSHCN  1.1. Care models and approach:  1.1.1. Health Care Networks: Integration and coordination of the different health service components: (1) population; (2) operational structure of the networks (primary, secondary, and tertiary care points, logistics systems, governance systems, support systems); (3) health care models;  1.1.2. Integrated Health Service Networks, a strategy to progressively reduce fragmentation of health services;  1.1.3. Comprehensive Health Care Model: based on a comprehensive approach to the human being, considering that the health/disease process is shaped by a combination of areas that condition the individual's experience of well-being;  1.1.4. Comprehensive Person-Centered Care Strategy, for promotion, prevention, and management of chronicity in the context of multimorbidity:  1.2. Comprehensive care model for CYSHCN and their families  1.2.1. Care focused on CYSHCN and families: CYSHCN, family, and/or primary caregiver are at the core of an individualized, flexible care plan, according to case complexity;  1.2.2. Assessment of care complexity and organization: determining complexity level allows, among other things, the provision of comprehensive care according to individual needs, organization of care, and optimizing of resources focused on each CYSHCN and family;  1.2.3. Management of CYSHCN care: care management is carried out by a nurse, with care centered on CYSHCN and family; management is comprehensive, flexible, dynamic, continuous, compassionate, coordinated, and culture-sensitive, to deliver safe, relevant, effective care to patient.  1.3. Registration and Monitoring System  1.3.1. CYSHCN Registry: to identify CYSHCN in a given territory and generate coordinated strategies;  1.3.2. Care management assessment: criteria to be considered when assessing CYSHCN care include coverage of all CYSHCN, monitoring of recovery and rehabilitation process (specialized care) and timely diagnostic assessment performed at first health check-up. |
| Title V Maternal and Child Health Services Block Grant to States Program Guidance and Forms for the Title V Application/Annual Report.  United States, 2023. | Each year, at least 30% of federal Title V funds must be used for services for CYSHCN, which include provision and promotion of coordinated, family-centered, community-based care (including care coordination services) for CYSHCN, facilitating the development of community-based service systems for these patients and their families. |
| Children and young people’s continuing care national framework.  United Kingdom, 2016. | Steps in the continuing care process:  Step 1: Identification; Step 2: Evaluation; Step 3: Recommendation; Step 4: Decision; Step 5: Reporting; Step 6: Organization of care; Step 7: Revision. |
| Progress, Persistence, and Hope: Building a System of Services for CYSHCN and Their Families.  United States, 2022. | Blueprint for Change focuses on four critical areas to ensure that CYSHCN can thrive: health equity, family and child well-being and quality of life, access to services, and financing of services. |
| Linee di indirizzo per la promozione e il miglioramento della qualità, della sicurezza e dell'appropriatezza degli interventi assistenziali in area pediatrico-adolescenziale.  Italy, 2017. | Comprehensive care comprising hospital and community, ensuring universal care provision.  All hospital units must provide clinical care documented and verified by indicators of treatment processes and results.  Health professionals involved in the treatment process must have skills to create partnerships with parents and family members; positively recognize cooperative behaviors in families and explain the consequences resulting from inappropriate behaviors; involve patients and family members in decisions related to the care pathway.  Pediatricians play a role in home monitoring and in the success of “self-management” of the disease, as well as in the treatment of any pathologies. This approach can ensure adequate home management, helping prevent unnecessary hospitalizations. |
| Desospitalização: reflexões para o cuidado em saúde e atuação multiprofessional.  Brazil, 2020. | The Health Care Network (RAS) provides coordination between levels of care, with the support of professionals such as doctors, nurses, social workers, physiotherapists, and psychologists for continuous monitoring, in order to use the Singular Therapeutic Project (PTS) as a management tool for individualized home care, prioritizing quality of life and shared decisions with families. |
| Entlassung von Kindern mit komplexen Versorgungsbedarfen von der neonatologischen Intensivstation/ Alemanha, 2024 | The aftercare process comprises six steps:  (1) Admission: assessment of medical and social support needs arising from complex conditions that may place a burden on the family. (2) Evaluation. (3) Planning: manager, family, and staff assess medical, therapeutic, social and psychological needs. (4) Implementation. (5) Monitoring. (6) Individual goals are adjusted as necessary, and achievement of goals is reviewed from several perspectives. |
