# Supplementary material for: Public health policies for children and youth with special health care needs in OECD member countries and Brazil: A scoping review
Source: PLoS One. 2026 Sep 18;21(9):e0326645. doi: 10.1371/journal.pone.0326645 (PMC13588354; doi:10.1371/journal.pone.0326645)
Supplement: Supplementary file 3 [file pone.0326645.s003.docx]

S3 Table. Key features of public health policies.

| **Country** | **Title, year** | **Description** | **Coordination mechanisms** | **Funding** | **Comprehensiveness of care** |
| --- | --- | --- | --- | --- | --- |
| Germany | Sozialmedizinische Nachsorge für schwer und chronisch kranke Kinder: Eine neue Leistung der Krankenkassen, 2009.  Entlassung von Kindern mit komplexen Versorgungsbedarfen von der neonatologischen Intensivstation, 2024. | Hospital-to-home transition management service, organization and coordination of outpatient therapies, medical visits and training, and rehabilitation services. Accompanies patients' families in the first weeks at home, provides emotional relief, and helps them cope with daily life. | A case manager organizes and coordinates outpatient care and all external partners in the network. | Medical-social follow-up is a mandatory service provided by health insurance companies, which fund medical and social care. | No, for not including health promotion measures, early disease detection, or prevention of risk factors, and for not providing follow-up for CYSHCN from birth throughout their lives. |
| Chile | Norma Técnica para la Supervisión de Salud Integral de Niños y Niñas de 0 a 9 Años en la Atención Primaria de Salud – Actualización 2021 (Volumen 3).  Orientación técnica para el manejo integral de niños, niñas y adolescentes con necesidades especiales de atención em salud (NANEA), 2022 | Technical guidance to define a comprehensive care plan and the collaboration of different levels of care for CYSHCN, starting at the primary level, with referral and counter-referral across levels. | Primary health care provides biopsychosocial follow-up of families under a comprehensive approach, to promote integration with secondary and tertiary levels, for best input from each level for CYSHCN.  Focus on family and community, to promote continuity of care. Social determinants of health, biopsychosocial risk factors, and health conditions and their consequences are addressed in a balanced approach. | Health services are provided through a public health system. | Yes, because a comprehensive approach is adopted from primary to the secondary and tertiary levels, for best benefit from each level for CYSHCN and their families. Prevention, health promotion, and continuity of care are addressed. Care network resources are optimized, involving family and community under a biopsychosocial approach combining cultural and territorial significance. |
| United States | Title V MCH Services Block Grant, 2024.  https://mchb.hrsa.gov/programs-impact/focus-areas/children-youth-special-health-care-needs-cyshcn;  https://mchb.hrsa.gov/programs-impact/title-v-maternal-child-health-mch-services-block-grant  Progress, Persistence, and Hope: Building a System of Services for CYSHCN and Their Families, 2022.  Title V Maternal and Child Health Services Block Grant to States Program Guidance and Forms for the Title V Application/Annual Report, 2023. | Strategic structural plan of a service system for CYSHCN focused on 4 critical areas: (1) health equity, (2) family and child well-being and quality of life, (3) access to services and support, and (4) funding for services.  Funding provides better access to coordinated family-centered, community-based, care systems. | Each state and jurisdiction supports and promotes development and coordination of family-centered, community-based, culturally appropriate care systems. | States apply for funding each year.  Federal government uses a formula to determine grant amount, based on size and needs of the state's population.  States and jurisdictions must match every US$4 of federal Title V funds they receive with at least US$3 of their own. | For the development of the MCHBlock grant application, states are encouraged in their program planning to ensure access to the comprehensive services needed for CYSHCN and families through public health services. |
| Poland | Uprawnienia szczególne, 2004  https://www.nfz.gov.pl/dla-pacjenta/prawa-pacjenta/uprawnienia-szczegolne/ | Law issued on August 27, 2004, guarantees special health services (*e.g.*, general anesthesia for dental services), prenatal diagnosis, specialized outpatient health care services and hospital treatments, psychological support, therapeutic rehabilitation, provision of medical devices, palliative care, and additional medical devices. | Coordinated care at the primary level is included in the list of basic health care services guaranteed by the Minister of Health Regulation, for faster, more effective diagnosis and treatment of chronic diseases (cardiological, pulmonary, endocrine, and renal), also facilitating access to specialist physicians. | Coordinated care services carried out with public funds through a contract with primary health care provider. | No details provided. |
| United Kingdom | Children and Young People’s Continuing Care National Framework, 2016 | Guidance tool combining resources, planning, coordination and support to assess the needs of CYSHCN that cannot be met solely by existing universal or specialist services. | A coordinator is responsible for ensuring an effective link with local authority and other partners, as well as ensuring effective management of the process, based on input from multiple professionals, focusing on results. | Families of eligible children and adolescents are entitled to a personal health budget funded by the National Health Service. | Services should aim to be integrated or aligned with other relevant services such as primary care, educational services and social care. |
| Italy | Linee di indirizzo per la promozione e il miglioramento della qualità, della sicurezza e dell'appropriatezza degli interventi assistenziali in area pediatrico-adolescenziale, 2017 | Guidelines to reduce the burden on families, improve quality of life, make health services more effective and efficient in terms of prevention and care, and ensure greater uniformity and equity of access. | Level I Centers should provide therapies in collaboration and coordination with the pediatric referral center.  Level II Centers should ensure multidisciplinary coordination of specialized diagnostic, therapeutic, and rehabilitation interventions through formal collaboration procedures involving departments within the same hospital and/or other associated facilities. | No details provided. | Service delivery is interconnected, from local level to specialized centers, including emergency care, to avoid current duplications, deficiencies, and inadequate access to emergency room.  The comprehensiveness of care, supported by a network of organizations that provide or arrange for the provision of equitable and comprehensive health services to users, is the basis of dehospitalization, as it presupposes formation and articulation of horizontal relationships between points of care, aiming at quality and patient-centered care. |
| Brazil | Desospitalização: reflexões para o cuidado em saúde e atuação multiprofissional, 2020 | Guideline for safe, responsible dehospitalization, based on a meticulous planning process, to prevent unnecessary readmission, supporting primary care teams and ensuring agility in the discharge to home and reintegration into Health Care Networks (RAS). | Care strategies for planning the discharge of CYSHCN, such as the Customized Therapeutic Project, are coordinated by one or more professionals tasked with bringing together multidisciplinary efforts to design the project and act as a link between hospital team, territory team, and family. | No details provided. | The comprehensiveness of care, supported by a network of organizations that provide or arrange for the provision of equitable, comprehensive health services, is the basis for dehospitalization, as it presupposes formation and articulation of horizontal relationships between points of care, aiming at quality and patient-centered care. |
