# Supplementary material for: Public health policies for children and youth with special health care needs in OECD member countries and Brazil: A scoping review
Source: PLoS One. 2026 Sep 18;21(9):e0326645. doi: 10.1371/journal.pone.0326645 (PMC13588354; doi:10.1371/journal.pone.0326645)
Supplement: Supplementary file 6 [file pone.0326645.s006.docx]

S1 Checklist. Manual search of websites

Search expression *children and youth with special health needs and health policies* was applied to first ten pages:

a) World Health Organization (WHO);
b) MiNDbank database of the WHO;
c) United Nations Children’s Fund (Unicef);
d) Maternal, Newborn, Child and Adolescent Health and Ageing data portal of the WHO;
e) Organization for Economic Cooperation and Development (OECD)
f) European Observatory on Health Systems and Policies;
g) Guidelines International Network;
h) US National Guideline Clearinghouse;
i) Google Scholar;
j) Research Gate;
k) Health Systems Evidence, Cochrane Library;
l) Center for Reviews and Dissemination;
m) Health Evidence;
n) Epistemonikos;
o) Catalogue of Theses and Dissertations of the Coordination for the Improvement of Higher Education Personnel (CAPES), on first ten pages with the expression *crianças e adolescentes com necessidades especiais de saúde e políticas públicas*, using the filters *Ciências da Saúde* for greater area of knowledge and *saúde pública e saúde coletiva* for area of knowledge;
p) Brazilian Digital Library of Theses and Dissertations, using the expression *crianças e adolescentes com necessidade especial de saúde*;
q) Open Access Theses and Dissertations (OATD), using the expression *children and youth with special health needs and health policies*;
r) ProQuest Dissertations & Theses Global (PQDT), using the expression *“children and youth with special health needs” and* *health policies*;
s) Open Access Scientific Repository of Portugal (RCAAP) using the expression *crianças com necessidades especiais de saúde*;
t) websites of ministries of health (or their equivalents) of member-countries of the OECD and Brazil;
u) Portal Periódicos CAPES (Brazil), using the expression *“children and youth with special health needs” and health policies*;
v) Biblioteca Virtual de Saúde (Virtual Health Library) of the Brazilian Ministry of Health.
