# Supplementary material for: Public health policies for children and youth with special health care needs in OECD member countries and Brazil: A scoping review
Source: PLoS One. 2026 Sep 18;21(9):e0326645. doi: 10.1371/journal.pone.0326645 (PMC13588354; doi:10.1371/journal.pone.0326645)
Supplement: Supplementary file 7 [file pone.0326645.s005.docx]

**Peer Review of Electronic Search Strategies (PRESS) guideline checklist.**
